# Ascending Aortic Coarctation - an Atypical Location in a Non-Takayasu Arteritis Female Patient

**DOI:** 10.21470/1678-9741-2022-0268

**Published:** 2023-06-14

**Authors:** Emre Oteyaka, Okan Eren Kuguoglu, Gizem Sari, Mehmet Turan Basunlu, Mehmet Sait Dogan, Elif Calis, Aykun Hakgor, Halil Turkoglu, Murat Ugurlucan

**Affiliations:** 1 Department of Cardiovascular Surgery, Faculty of Medicine, Istanbul Medipol University, Istanbul, Bagcilar, Turkey; 2 Department of Pediatric Cardiology, Faculty of Medicine, Istanbul Medipol University, Istanbul, Bagcilar, Turkey; 3 Department of Radiology, Faculty of Medicine, Istanbul Medipol University, Istanbul, Bagcilar, Turkey; 4 Department of Pathology, Faculty of Medicine, Istanbul Medipol University, Istanbul, Bagcilar, Turkey; 5 Department of Cardiology, Faculty of Medicine, Istanbul Medipol University, Istanbul, Bagcilar, Turkey; 6 Department of Cardiology, Faculty of Medicine, Istanbul Medipol University, Istanbul, Bagcilar, Turkey

**Keywords:** Aortic Coarctation, Atherosclerosis, Takayasu Arteritis

## Abstract

Coarctation of the aorta is a well-known congenital cardiovascular disorder that typically occurs within proximity to the ductus arteriosus. The ascending aorta, distal descending aorta, and abdominal aorta are segments which are prone to development of an atypical coarctation. The etiologies of atypical cases are usually associated with various types of vasculitis syndromes or underlying genetic disorders. In this report, we present a 24-year-old female patient with an ascending aortic coarctation which developed secondary to an atherosclerotic process.

**Table:** 

Abbreviations, Acronyms & Symbols	
AR	= Anterior right
COA	= Coarctation of the aorta
FRP	= Foot right posterior
HLA	= Head left anterior
PL	= Posterior left

## INTRODUCTION

Coarctation of the aorta (CoA) is a congenital cardiovascular anomaly that typically occurs within the vicinity of the ductus arteriosus. It has a prevalence of four in 10,000 live births^[1,2]^. Most of CoA cases are congenital, and the acquired form of the disease is rare. The majority of acquired CoAs are due to inflammatory diseases of the aorta^[3]^. Patients may present to the clinic with symptoms of hypertensive headaches, epistaxis, and aortic dissection. The choice of surgical or radiologic interventional treatment depends on the size and anatomy of the lesion.

We report the presentation and treatment of a 24-year-old woman with an ascending aortic coarctation secondary to an atherosclerotic process.

## CASE PRESENTATION

A 24-year-old female patient referred to an institutional hospital with complaints of paresthesia of the right arm, fatigue, and exertional dyspnea. Her past medical history was unremarkable with a family history of hypertension and diabetes. On physical examination, patient’s heart was hyperdynamic, there was a strong systolic murmur over the upper border of the sternum. The right carotid artery and the upper extremity were pulseless. Echocardiography indicated significant supravalvular ascending aortic stenosis, left ventricular hypertrophy, left atrial dilatation, and moderate mitral regurgitation.

**Fig. 1:**
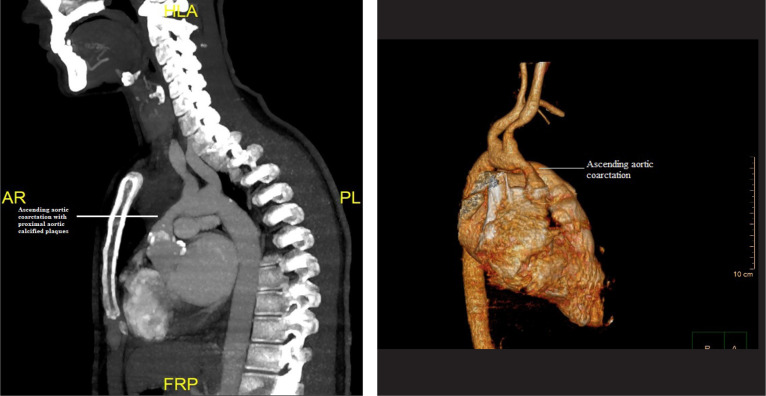
**A and B** - Computerized tomography angiography of the aorta indicating ascending aortic coarctation with proximal aortic calcified plaques. AR=anterior right; FRP=foot right posterior; HLA=head left anterior; PL=posterior left.

A computerized tomography angiography was conducted, which indicated occlusion of the brachiocephalic trunk and ascending aortic coarctation together with calcified plaques throughout the coronary arteries (Figures 1A and B). A supracoronary ascending aortic replacement using a Dacron® graft was planned for the patient. The patient was scheduled for corrective surgery following the consent of the family after being informed about the risks and benefits of the treatment in details.

### Surgical Treatment

**Fig. 2:**
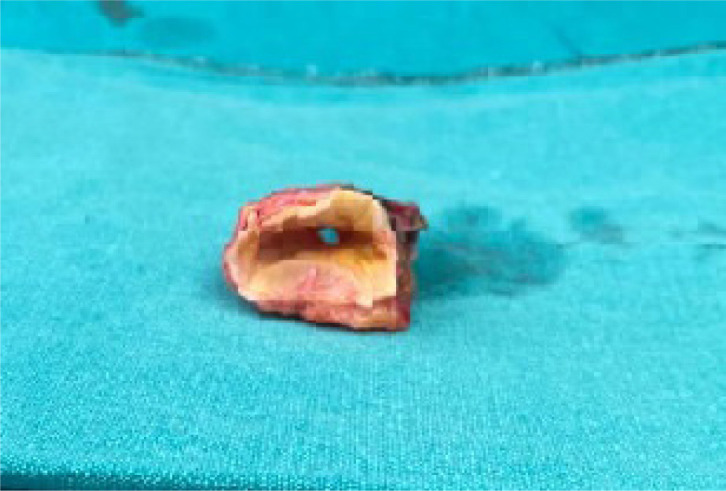
Excised ascending aorta showing significant narrowing.

A median sternotomy was performed with general anesthesia. The pericardium was opened and narrowing of the ascending aorta was observed. Right femoral artery was prepared with groin incision. The left common carotid artery, right femoral artery, and atrial two-stage cannulations were performed, and cardiopulmonary bypass was initiated. Cardiac arrest was achieved with antegrade cold blood cardioplegia. The narrow portion of the ascending aorta was resected (Figure 2). The patient underwent supracoronary ascending aortic and hemiarch replacement with a 28 mm Dacron® tube graft (Intergard, Maquet Getinge Group, Goteborg, Sweden). Clamps were removed after air evacuation. Patient was weaned off cardiopulmonary bypass with 5 mcg/kg/min dopamine support, decannulated, and operation was finalized following standard measures. The total cross-clamping and cardiopulmonary bypass times were 44 minutes and 58 minutes, respectively. The patient was transferred to the intensive care unit and was extubated 16 hours following the operation. After three days of follow-up in the intensive care unit, the patient was transferred to the ward and discharged from the hospital after spending seven days in good condition. The patient was followed actively and showed normal myocardial function with considerable weight gain and growth.

**Fig. 3:**
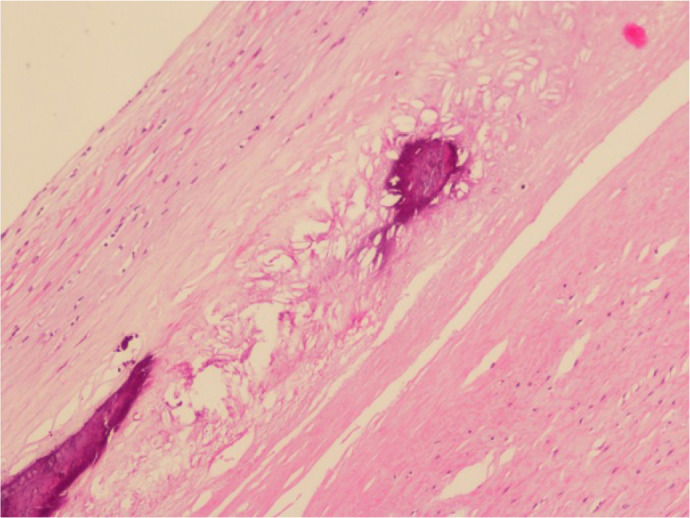
Histopathologic examination of the ascending aorta with calcified atherosclerotic plaque and thick fibrous cap (haematoxylin & eosin × 100).

The histopathologic examination of the excised ascending aorta indicated calcified atherosclerotic plaques with thickened fibrous caps (Figure 3) indicative of an atherosclerotic process, interestingly, rather an inflammatory vasculitis process such as Takayasu arteritis especially considering the clinical features of the patient.

## DISCUSSION

Abnormalities in development of pharyngeal arches and their arterial system during the embryonic period can lead to various aortic anomalies including CoA^[1]^. Although most of the cases are related with developmental abnormalities, autoimmune vasculitis syndromes and atherosclerotic processes may also be associated with adult onset atypical coarctation. Most cases presenting with acquired CoA can be attributed to vascular inflammatory syndromes such as Takayasu arteritis, which carries an increased incidence in the female population^[4]^. Takayasu arteritis is an idiopathic granulomatous vasculitis effecting the aorta and its branches. Inflammation and intimal proliferation leading to thickening of the vascular walls along with stenosis or occlusions, thrombosis, and the destruction of the elastic and muscular layers can lead to aneurysm formation and dissection along the lining of vessels^[5]^.

Clinical picture of a patient presenting with Takayasu arteritis includes carotid artery stenosis, claudication, ocular disturbances, central nervous system abnormalities, and weakening of pulses. The diagnosis can be confirmed with observation of large vessel wall abnormalities such as stenosis, aneurysms, and occlusion on imaging. A histopathological examination can also confirm the diagnosis, with mononuclear cells, predominantly lymphocytes, histiocytes, macrophages, and plasma cells with giant cells and granulomatous inflammation typically seen in the media. Destruction of the elastic lamina and the muscular media can present as aneurysmal dilation of the vessel affected. Vascular lumen is compromised due to progressive inflammation and dense scarring reaching the adventitia with intimal proliferation contributing to the development of stenotic arterial lesions^[6]^.

Although rare, chronic inflammatory processes such as atherosclerosis is also among the etiologies which can lead to atypical CoA. This arises when a calcified plaque along the endothelial lining of the artery induces an acquired coarctation due to significant luminal stenosis. However, such cases mainly present with localization of the coarctation at the juxta-renal and infrarenal segments of the aorta^[7]^. Other etiologic connective tissue abnormalities, such as William’s syndrome, could be another cause of CoA. In William’s syndrome, elastin gene mutation causes proliferation of smooth muscle cells and fibroblasts, decreases arterial elasticity with irregular arrangement of short elastic fibers, and causes luminal stenosis with medial thickening of the muscular layer of large arteries, leading to CoA^[5-7]^. The prevalence of CoA in patients with Williams syndrome was presented as 18% by Collins et al.^[8]^. Atherosclerotic process is also among etiologies and in this condition the localization of the coarctation is mainly at the juxtorenal and suprarenal segments of the aorta^[9]^. Investigation of underlying factors which may have led to the formation of the atherosclerosis along the ascending aorta such as dyslipidemia, smoking, and hypertension, are not sufficient to justify the pathogenesis in such cases^[10]^. Disturbances of calcium metabolism and infectious agents affecting the vessel wall could be responsible for atherosclerotic processes of this extent, but further studies are required to determine the etiology^[10]^.

Acquired CoA due to atherosclerosis can present with blood pressure gradient between the upper and the lower extremities, visceral and peripheral ischemia, heart failure due to increased afterload, or hypertension due to renal ischemia. Clinical findings may include claudication of lower limbs bilaterally, renovascular hypertension, abdominal angina, weight loss, microvascular embolization in distal organs, impaired renal function, and subsequently end-stage renal disease^[9]^.

Failure to identify the disease may result in life-threatening renal and visceral complications and irreversible organ damage due to delay between the presentation of symptoms and initiation of treatment. Therefore, early diagnosis and treatment of acquired CoA may sometimes be lifesaving. Treatment modalities include aortic balloon and stent dilatation, transaortic thromboendarterectomy, and various bypass procedures depending on the localization of the segmental stenosis^[7-13]^.

## CONCLUSION

Since our patient was a 24-year-old woman presenting with an acquired ascending aortic coarctation, Takayasu arteritis was suspected as the underlying etiology and the excised ascending aorta was sent for histopathologic examination. The results of the pathology indicated an atherosclerotic plaque formation along the tunica intima layer of the aorta at an atypical site for CoA (Figure 3). Even though it is often difficult to distinguish the scarring stage of Takayasu arteritis from arteriosclerosis, the former shows tearing and fibrosis of the medial elastic fibers, fibrous thickening of the adventitia, and characteristic cell infiltration^[4-6]^, and although limited to one case, the findings published by Yoshida M. et al.^[14]^ raise the possibility that long-term persistent and severe inflammation of Takayasu arteritis alone is not sufficient to induce the progression of arterial damage presenting as atherosclerosis. However, histopathological examination of the surgically excised tissue was negative for Takayasu arteritis and indicated atherosclerosis alone as the cause for the coarctation. Further investigation into cases of atherosclerosis in patients with ascending aortic coarctation, which is an atypical site of coarctation, in non-Takayasu arteritis patients is required to determine the underlying etiologies which resulted in such a case.

**Table:** 

Authors’ Roles & Responsibilities	
EO	Drafting the work or revising it critically for important intellectual content; final approval of the version to be published
OEK	Drafting the work or revising it critically for important intellectual content; final approval of the version to be published
GS	Substantial contributions to the conception or design of the work; final approval of the version to be published
MTB	Substantial contributions to the conception or design of the work; final approval of the version to be published
MSD	Substantial contributions to the acquisition, analysis, or interpretation of data for the work; final approval of the version to be published
EC	Substantial contributions to the acquisition, analysis, or interpretation of data for the work; final approval of the version to be published
AH	Substantial contributions to the acquisition, analysis, or interpretation of data for the work; final approval of the version to be published
HT	Agreement to be accountable for all aspects of the work in ensuring that questions related to the accuracy or integrity of any part of the work are appropriately investigated and resolved; final approval of the version to be published
MU	Drafting the work or revising it critically for important intellectual content, agreement to be accountable for all aspects of the work in ensuring that questions related to the accuracy or integrity of any part of the work are appropriately investigated and resolved; final approval of the version to be published
